# Nutrition status among adults in the face of household food insecurity: a cross-sectional study in five different regions in senegal

**DOI:** 10.1186/s40066-026-00643-7

**Published:** 2026-06-14

**Authors:** Babatunde Owolodun, Sonja Merten

**Affiliations:** 1 Swiss Tropical and Public Health Institute, Basel, Switzerland; 2 University of Basel, Basel, Switzerland

**Keywords:** Food security, Malnutrition, Nutrition transition, Body mass index, Senegal

## Abstract

**Background:**

Household food insecurity remains a critical public health issue in low- and middle-income countries, contributing to undernutrition and overweight. Senegal is undergoing a nutrition transition, with regions experiencing varying degrees of food insecurity and nutritional outcomes. This study examines the association between household food insecurity and adult nutritional status across five ecologically distinct regions of Senegal.

**Method:**

A cross-sectional study was conducted from April to June 2024 involving 1,965 adults from 1,253 households in Boundou, Bedik/Bandafassi, Senegal River Valley, Lower Casamance, and Dakar regions. Household food insecurity was assessed using the validated Household Food Insecurity Access Scale, while body mass index (BMI) was calculated from measured weight and height. Associations between food insecurity, socioeconomic factors, and BMI categories were analyzed using multilevel ordinal logistic regression with region-stratified analyses conducted to evaluate context-specific relationships.

**Results:**

Findings revealed significant geographic variation in food security and nutritional outcomes. Over 50% of households in Boundou and Bedik/Bandafassi were severely food insecure, while Lower Casamance had the highest food security levels. Underweight was most prevalent in food-insecure rural regions like Boundou, whereas overweight and obesity were concentrated in urban areas like Dakar and Lower Casamance. Food insecurity was significantly associated with higher BMI in Boundou and overweight in Dakar, supporting the food insecurity-obesity hypothesis. However, no significant association emerged between food insecurity and underweight in the overall model, suggesting that broader ecological and health-related factors may drive undernutrition.

**Conclusion:**

The study highlighted the context-specific and regionally differentiated relationships between food insecurity and nutritional status in Senegal. These findings underscore the need for locally tailored, multisectoral interventions that address the dual burden of malnutrition by improving both food access and diet quality.

**Supplementary Information:**

The online version contains supplementary material available at 10.1186/s40066-026-00643-7.

## Background

Household food insecurity is a core indicator of nutritional status and remains a major public health concern, particularly in low- and middle-income countries where access to sufficient, safe, and nutritious food is often limited [1]. Food insecurity is characterized by limited or uncertain access to adequate and nutritious food, which has profound implications for dietary intake, nutritional status, and overall health outcomes [2]. The concept of food insecurity encompasses challenges related to both the quantity and quality of available food, as well as uncertainty about food supply [3] and experiences of hunger, defined as an uncomfortable or painful physical sensation caused by insufficient consumption of dietary energy [4]. Adequate nutrition is essential for maintaining good health and preventing diet-related diseases. However, limited food availability and accessibility have been linked to poor nutritional outcomes, including both undernutrition and overweight/obesity, creating a paradoxical relationship between food insecurity and malnutrition [5, 6].

Globally, food insecurity shows significant regional disparities, with Africa being the most affected region with approximately 20% of its population facing hunger in 2021 compared to 9.1% in Asia, 8.6% in Latin America and the Caribbean, and less than 2.5% in Northern America and Europe [7]. While food availability and access continue to pose major obstacles to realizing the right to food in Africa, the issue is further intensified by the poor quality and nutritional adequacy of the food that is accessible, with 78% of Africa’s population unable to afford a healthy diet, contributing to widespread nutrient deficiencies and a growing burden of diet-related diseases [8]. In sub-Saharan Africa, food insecurity is frequently influenced by economic disparities, environmental challenges, and sociocultural norms that shape dietary behaviors and overall health [9]. In addition, urbanization has also influenced food choices, often through increased access to processed and energy-dense foods, reduced physical activity, and shifts away from traditional diets, resulting in undernutrition, thereby contributing to the double burden of malnutrition, where undernutrition and obesity coexist within the same communities or households [10, 11].

Conversely, in low and middle-income countries (LMICs), food insecurity has been associated with poor dietary intake, malnutrition, and underweight (as opposed to obesity as seen in high-income countries). However, as many low- to middle-income countries undergo a nutrition transition, a phenomenon marked by shifts in dietary patterns and nutrient intake, including increased consumption of processed and energy-dense foods as populations adopt modern lifestyles during economic and social development (9), the diet and health outcomes linked to food insecurity are increasingly resembling those observed in high-income nations [10, 11].

Food insecurity can significantly affect individuals’ nutritional and overall health status, with the nature of these effects differing between high-income and low- to middle-income countries. In high-income countries, food insecurity among children is often linked to developmental delays, both physical and psychological, as well as reduced academic performance [2, 12]. Among adults, food insecurity tends to be associated with poor dietary choices that may lead to overweight and obesity (especially among women), poor physical health, depression, and a higher risk of chronic, diet-related illnesses, including diabetes and cardiovascular diseases [13, 14]. While the immediate impact of household food insecurity often manifests as undernutrition, particularly among children, emerging evidence suggests a complex relationship between food insecurity and various forms of malnutrition in adults, including overweight and obesity (11, 17). However, the mechanisms underlying these associations are complex and context-specific, influenced by factors such as socioeconomic status, household composition, and regional disparities (5). For instance, food insecurity may lead to reliance on energy-dense, nutrient-poor foods, contributing to overweight and obesity, while simultaneously limiting access to diverse diets, resulting in undernutrition (8, 17).

Senegal, like many other countries in sub-Saharan Africa, faces the challenge of widespread food insecurity, with varying levels of severity across different regions [15]. According to the Cadre Harmonisé national food security analysis, acute food insecurity intensifies during the lean season, when about 1.3 million people in Senegal were estimated to face acute food insecurity during the 2025 lean season [16]. More recent reports from the World Food Programme indicate that around half a million people in Senegal were classified as facing Crisis-level food insecurity in early 2026, with this figure projected to nearly double to close to 1 million people during the June to August 2026 lean season [17]. Urban and rural populations exhibit varying degrees of food security, with rural areas experiencing more significant constraints in food access due to seasonal variations, reliance on subsistence agriculture, and economic instability, which contribute to unequal nutrition outcomes among adults across the country [18, 19]. Furthermore, climate change has affected food production through frequent heatwaves, droughts, particularly in the northern part of Senegal, as well as poor land management and other factors, such as deforestation in the south [20].

This study is necessary to address critical gaps in understanding how food insecurity impacts adult nutritional outcomes across diverse geographic and socio-economic contexts within Senegal, particularly in relation to urban–rural disparities. While national-level data often provide valuable overviews, they tend to obscure localized variations in food access, dietary patterns, and health outcomes that are shaped by both structural and environmental factors. Rural areas, for instance, frequently contend with greater barriers to food access due to seasonal agricultural cycles, limited infrastructure, and heightened vulnerability to climate-related shocks. In contrast, urban populations, though closer to food markets, may face economic constraints that push them toward consumption of inexpensive, calorie-dense, but nutrient-poor foods. These distinct dynamics influence how food insecurity manifests and affect nutritional status in different ways, contributing to the dual burden of malnutrition in context-specific forms. By exploring five geographically and socioecologically diverse locations, this study provides critical empirical evidence to inform more nuanced, regionally tailored interventions. The results can help guide policymakers in designing targeted, context-aware strategies that address both immediate food access concerns and long-term nutritional health outcomes, thereby supporting Senegal’s broader goals for sustainable food systems and equitable health development. Given the above considerations, the aim of the current study was to investigate the possible association between food insecurity and body mass index (BMI) in five locations in Senegal.

## Methods

### Study setting and context

**Fig. 1:**
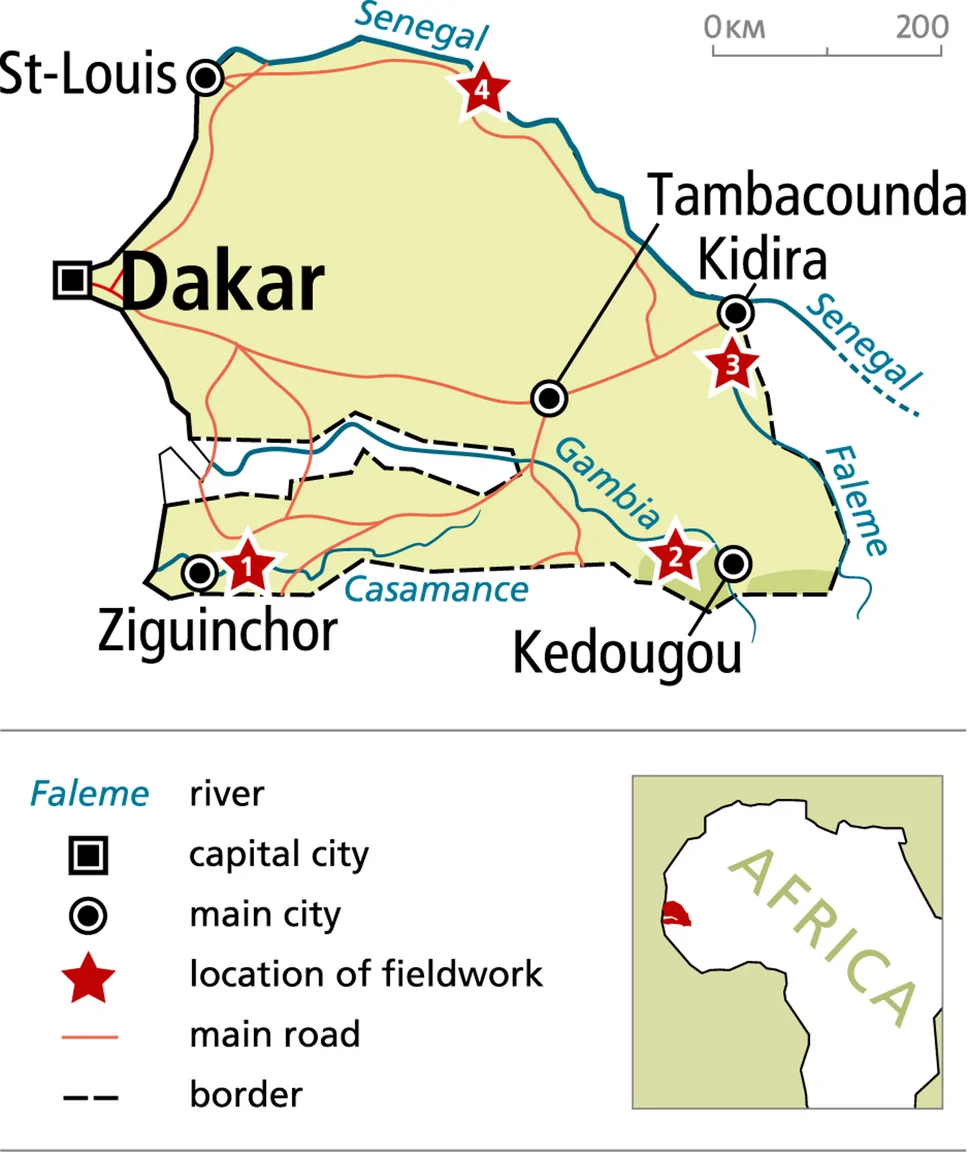
Map of the study areas in Senegal. (Source: own work)

Senegal is a West African country bordered by Mauritania to the north, Mali to the east, Guinea and Guinea-Bissau to the south, and the Atlantic Ocean to the west [21]. This study was conducted in five distinct regions of Senegal (Fig. 1): Tambacounda (Boundou), Kedougou (Bedik Country), Saint-Louis (Senegal River Valley), Ziguinchor (Lower Casamance), and Dakar. These regions were selected based on their diverse food systems. Lower Casamance is characterized by rice agriculture and fisheries within its floodplain landscape and is largely associated with Diola communities, while the Bedik Country includes Bedik and Peul populations whose livelihoods are linked to rainfed agriculture and agro-pastoralism in a semi-arid environment [22]. Boundou, located in a dryland area and predominantly inhabited by Peul and Jakhanke communities, is mainly pastoral, whereas the Senegal River Valley, which includes the major ethnic groups Peul, Soninké, Maures, and Wolof communities, integrates rainfed and industrial agriculture, agro-pastoralism, and fishing within its floodplain ecosystem [22]. Dakar, as an urban and ethnically diverse area, complemented the four rural sites.

### Study design and sampling

This cross-sectional household study was conducted among 1,965 adults from 1,253 households across five regions of Senegal between April 2024 and June 2024. A two-stage cluster sampling was conducted in five different regions, where approximately 44 enumeration areas were randomly selected in these regions using a probability proportional to size approach, with size defined as the number of households in each enumeration area. Households in the selected enumeration areas were listed out to ensure that the measures of size are fully updated. From the updated listing, 20 households were randomly selected, forming the survey clusters. The sample size was determined a priori based on expected between-region differences in Food Consumption Score (FCS), a key continuous indicator collected as part of the broader survey. Previous studies in comparable sub-Saharan African settings reported standard deviations below 20 points [23]. To ensure conservative estimates, we assumed a within-region standard deviation of 20 points and a design effect of 2.0 to account for cluster sampling, a minimum of 218 households per region was required to estimate the mean FCS with a margin of error of 3.75 points. This sample size provided 80% power at the 5% significance level to detect between-region differences of 7.5 points or greater. The achieved sample met these requirements and forms the basis of the present analysis examining household food insecurity and adult BMI. Although a priori sample size was calculated based on FCS, both BMI and HFIAS were planned core measures of the parent survey from the outset, and the achieved sample is well in excess of what is typically required to detect associations of moderate magnitude between household food insecurity and adult BMI in pooled multilevel analyses. In each household surveyed, at least one adult, both male and female, present at the time of the survey, had their anthropometric measurement taken, preferably the one responsible for the decisions around the family’s feeding.

### Measurements

The outcome of interest was Body mass index (BMI). During the visit, when food insecurity was assessed, we measured the weight and height of participants based on the standard protocols of WHO [24]. BMI, which is an indicator of the nutritional status of adults, reflects chronic energy deficiency and excess energy status, and was calculated by dividing an individual’s weight (kg) by height in metres squared (m²). The BMI was classified into four categories: Underweight (BMI < 18.5), Normal weight (BMI 18.5 to < 24.9), Overweight (BMI 25 to 29.9), and Obesity (BMI ≥ 30), in accordance with WHO classifications [24].

As the main exposure, data on household food insecurity were collected using the Household Food Insecurity Access Scale (HFIAS), originally developed by the USAID-funded Food and Nutrition Technical Assistance II project. Participants were asked to provide information on their household food status in the month before the survey. The original HFIAS was previously validated in several contexts, including the United States and in LMICs [25–27]. The HFIAS captures households’ behavioral and psychological manifestations of insecure food access. The HFIAS module covers a recall period of 30 days and consists of two types of questions: nine “occurrence” and nine “frequency-of-occurrence” questions, which produce a total score between 0 and 27, with higher scores indicating greater food insecurity [25]. Households were categorized into four levels of food insecurity (food secure, mildly, moderately, or severely food insecure) depending on the number of positive responses to questions related to severe conditions [25]. The Household Food Insecurity Access Scale was subsequently treated as a 2-category variable by dichotomizing the four categories into two distinctive categories: food secure (coded as 0) and food insecure (including mildly, moderately, and severely food insecure households, coded as 1).

A household wealth index, which is based on household assets and the quality of housing, was used as a proxy indicator for the socioeconomic status (SES) of households. This index is created by summing dummy variables derived from information collected on various factors, including housing quality (such as the materials used for flooring, walls, and roofing), availability of potable water, the type of toilet facilities, and ownership of durable goods and livestock (e.g., bicycle, television, radio, motorcycle, sewing machine, telephone, cars, refrigerator, mattress, and bed). The wealth variable categorized respondents into quintiles according to the household’s score on the Demographic and Health Survey (DHS) wealth index, which was based on the household’s amenities, assets, and living conditions [28].

### Data collection

Data were collected using tablets with the Open Data Kit software package, and the questions were translated into the main local language, French. A team of 20 local researchers, fluent in the locally spoken languages, was trained over a week in data collection methods, followed by a pilot study. The survey questionnaire included the Household Food Insecurity Access Scale (HFIAS), a previously validated tool. The household wealth index was constructed using items adapted from the Demographic and Health Survey (DHS) methodology and tailored to the local geographic context. Sociodemographic variables, including age, sex, and household characteristics, were collected using structured survey questions developed for the study. Anthropometric measurements (weight and height) were collected according to WHO standard protocols. Weight was measured in kilograms (kg) using a digital scale, and height was measured in meters (m) using a stadiometer. Weight measurements were taken with participants wearing light clothing and no shoes.

### Statistical analyses

Descriptive statistics were tabulated to describe the characteristics of households and individuals in each level of food security using means ± standard deviations and proportions with percentages for continuous and categorical variables, respectively. Multilevel mixed-effects ordinal logistic regression was conducted to explore the association of HFIAS and household characteristics with BMI status, accounting for the hierarchical structure of the data, with individuals nested within households and households nested within study areas. This approach was appropriate given the clustered sampling design and the potential correlation of observations within households and regions. Random intercepts were included at both the household and study area levels to account for unobserved heterogeneity. In this ordinal framework, BMI was treated as an ordered outcome across four categories (underweight, normal weight, overweight, and obesity), and associations are therefore described in terms of higher or lower BMI in the pooled analysis. In addition, separate multilevel logistic regression models were run for each BMI category (obesity, overweight, and underweight) to further explore outcome-specific associations. Crude and adjusted odds ratios (AOR) were reported for associations, and mixed-effects models were used to account for the clustering design. Unadjusted (crude) models were fitted using the same multilevel mixed-effects structure as the adjusted models but included only the exposure variable of interest and the outcome, without additional covariates.

The Akaike Information Criterion (AIC) was used to compare different models; the model with the lowest AIC was retained. Odds ratios with 95% confidence intervals (CIs) were calculated, and statistical significance was accepted at the 5% level of significance (*p* < 0.05). All adjusted models included age, sex, household wealth quintile, and daily household food expenditure as covariates. To determine whether the relationship between food insecurity and nutritional outcomes varied by geographic context, we tested for interaction between study area (region) and food insecurity using interaction terms in the pooled multilevel models for BMI, and each of its categories (underweight, overweight, and obesity) prior to conducting stratified analyses. The interaction was statistically significant for BMI, underweight, and overweight, but not for obesity. Based on these results, region-stratified models were conducted for BMI, underweight, and overweight to explore context-specific associations across the diverse socioecological settings. Because no statistically significant interaction was observed for obesity, only a pooled multilevel model was presented. All data were analyzed using Stata 18.0.

## Results

**Table 1:** Household-level sociodemographic characteristics (*N* = 1253 households)

Variable	Frequency	Percentage
Study area		
Boundou Bedik/Bandafassi Senegal River Lower Casamance Dakar	288 281 241 241 202	23.0 22.4 19.2 19.2 16.2
Ethnicity of household		
Peul Bedik Wolof Diola Jakhankes Serer Others	633 181 148 214 27 20 30	50.5 14.4 11.8 17.1 2.2 1.6 2.4
**Number of people residing in the household**		
1–5 6–10 11 and more	298 482 473	23.8 38.5 37.7
**Wealth quintile**		
Poor Poorer Middle Richer Richest	250 260 249 244 250	19.9 20.8 19.9 19.5 19.9
**Daily household food expenditure**		
<4000 CFA >4000 CFA	864 389	68.9 31.1
**Member of the household owning land**		
No Yes	749 504	59.8 40.2

Table 1 shows the household characteristics, which comprise 1,253 households from 5 different regions in Senegal. The ethnic distribution indicates that approximately 50% of the households surveyed belonged to the Peul ethnic group, followed by the Diola (17.1%), Bedik (14.5%), and Wolof (11.8%). Other ethnic groups, including Jakhankes (2.2%) and Serer (1.6%), were less represented, while 2.4% of households identified as belonging to other ethnicities. Household size varied, with 38.5% of households having 6–10 members and 37.8% having 11 or more members. Smaller households, comprising 1–5 members, accounted for 23.8% of the sample.

The wealth quintile distribution was relatively even across quintiles, the poorest and richest quintiles each comprised 19.9% of the sample, while the poorer (20.8%), middle (19.9%), and richer (19.5%) quintiles had comparable proportions. Daily household food expenditure was predominantly below 4000 CFA (68.9%), with only 31.1% of households spending more than 4000 CFA on food daily. Regarding land ownership, 40.2% of households reported having at least one member who owned land, while 59.8% did not.

**Table 2:** Distribution of household food security by study area

Household food security	Boundou *N* (%)	Bedik/Bandafassi *N* (%)	Senegal River *N* (%)	Lower Casamance *N* (%)	Dakar *N* (%)
**Food secure**	84 (29.2)	72 (25.6)	109 (45.2)	227 (94.2)	122 (60.4)
**Mildly food insecure**	17 (5.9)	15 (5.3)	10 (4.2)	7 (2.9)	19 (9.4)
**Moderately food insecure**	30 (10.4)	44 (15.7)	23 (9.5)	2 (0.8)	21 (10.4)
**Severely food insecure**	157 (54.5)	150 (53.4)	99 (41.1)	5 (2.1)	40 (19.8)
**Total (N)**	288	281	241	241	202

**Fig. 2:**
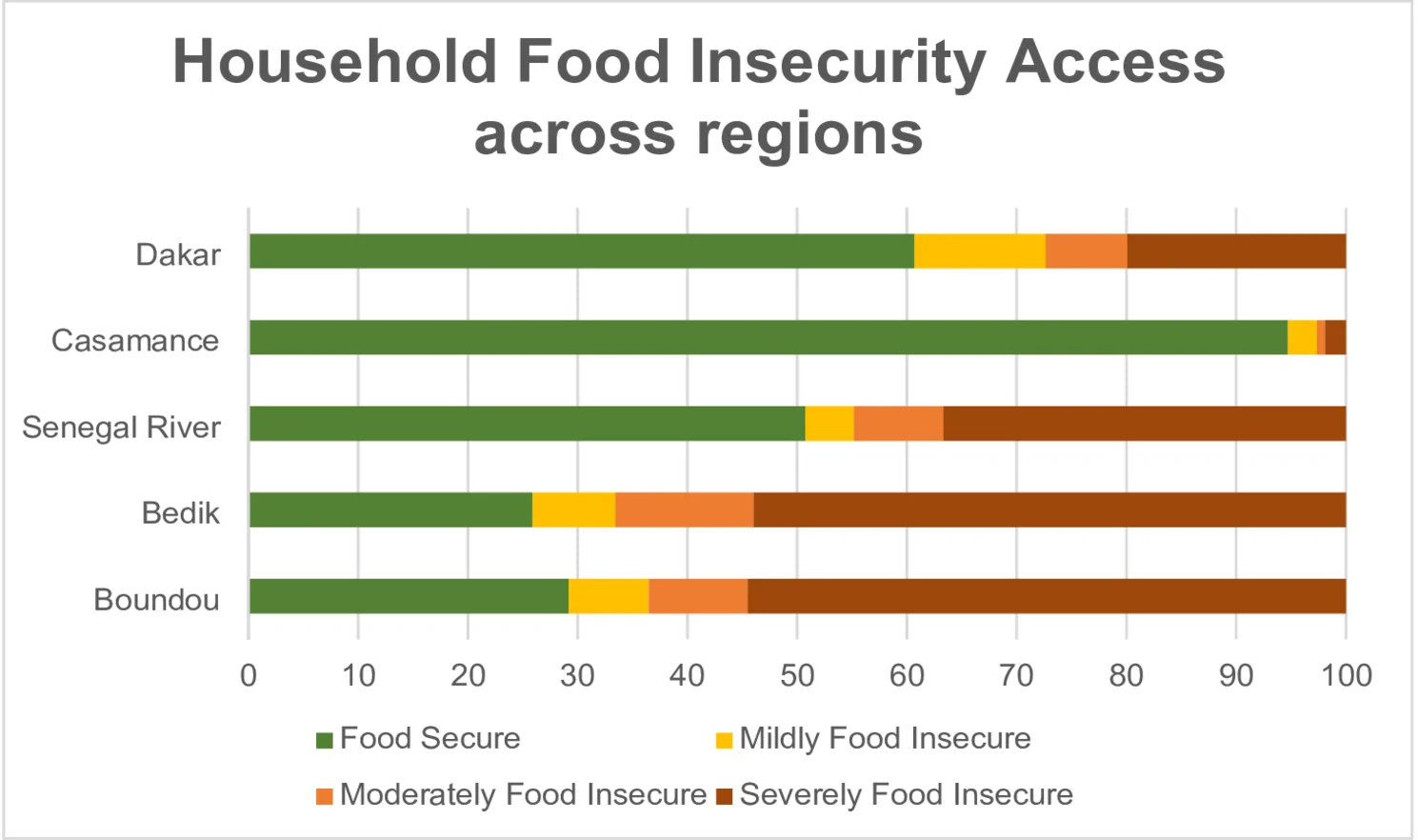
Household food insecurity level by region

Table 2 shows that the proportion of food-secure households varied significantly by region. Casamance had the highest proportion of food-secure households (94.2%), followed by Dakar (60.4%) and the Senegal River region (45.2%). In contrast, Boundou (29.2%) and Bedik/Bandafassi (25.6%) had the lowest proportions of food-secure households, indicating higher levels of food insecurity in these areas. Mild food insecurity was relatively low across all regions, with proportions ranging from 2.9% in Casamance to 9.4% in Dakar. Moderately food-insecure households were more prevalent in Bedik/Bandafassi (15.7%) and Boundou (10.4%), while Casamance had the lowest proportion (0.8%). Severe food insecurity was most prevalent in Boundou and Bedik/Bandafassi, where 54.5% and 53.4% of households, respectively, were severely food insecure as shown in Fig. 2. In the Senegal River region, 41.1% of households experienced severe food insecurity. Lower Casamance had the lowest prevalence of severe food insecurity (2.1%), aligning with its high proportion of food-secure households, while Dakar also had a lower prevalence of severe food insecurity (19.8%) compared to the most affected regions. Overall, the findings highlighted significant regional disparities in household food security, with Lower Casamance exhibiting the highest level of food security and Boundou and Bedik/Bandafassi experiencing the highest levels of severe food insecurity.

**Table 3:** Sociodemographic characteristics of adults in food secure and food insecure households

Variables	Food-secure households *n* (%)	Food-insecure households *n* (%)
**Sex**		
Men Women	374 (38.5) 598 (61.5)	368 (37.1) 625 (62.9)
**Mean age: 41.87 ± 16.43**		
**Age**		
20–39 40–59 60 and above	518 (53.3) 275 (28.3) 179 (18.9)	512 (51.6) 296 (29.8) 185 (18.6)

Table 3 presents the sociodemographic characteristics of adults in food-secure and food-insecure households. The mean age of respondents was 41.87 ± 16.43 years, with women constituting the majority in both food-secure (61.5%) and food-insecure (62.9%) households, while men accounted for 38.5% and 37.1%, respectively. The largest proportion of adults in both food-secure (53.3%) and food-insecure (51.6%) households fell within the 20–39 age bracket. The 40–59 age group comprised 28.3% of those in food-secure households and 29.8% in food-insecure households. Meanwhile, individuals aged 60 and above constituted the smallest proportion, accounting for 18.9% in food-secure households and 18.6% in food-insecure households.

**Table 4:** Nutritional Status by study area

BMI	Boundou *N* (%)	Bedik/Bandafassi *N* (%)	Senegal River *N* (%)	Lower Casamance *N* (%)	Dakar *N* (%)
Underweight	100 (22.6)	70 (17.5)	47 (12.3)	60 (14.2)	29 (9.1)
Normal weight	233 (52.7)	243 (60.8)	246 (64.2)	206 (48.8)	150 (47.2)
Overweight	74 (16.7)	59 (14.8)	70 (18.3)	96 (22.8)	93 (29.3)
Obesity	35 (7.9)	28 (7)	20 (5.2)	60 (14.2)	46 (14.5)

Table 4 presents the distribution of Body Mass Index (BMI) categories across the study regions. The proportion of underweight individuals classified varied by region, with the highest prevalence observed in Boundou (22.6%) and Bedik/Bandafassi (17.5%). The Senegal River region (12.3%), Casamance (14.2%), and Dakar (9.1%) had lower proportions of underweight individuals. The prevalence of overweight individuals was highest in Dakar (29.3%) and Casamance (22.8%), followed by Senegal River (18.3%), whereas Boundou (16.7%) and Bedik/Bandafassi (14.8%) had lower proportions. Obesity rates also varied across regions, with the highest percentages observed in Dakar (14.5%) and Casamance (14.2%). The lowest obesity prevalence was recorded in the Senegal River region (5.2%), followed by Bedik/Bandafassi (7%) and Boundou (7.9%).

**Table 5:** Crude and adjusted multilevel regression model for the association between household characteristics, household food insecurity, and BMI

Variables	Unadjusted OR (95% CI)	*P* value	Adjusted OR (95% CI)	*P* value
**Age**	1.14 (1.10, 1.18)	**0.000**	1.13 (1.09, 1.17)	**0.000**
**Sex**				
Male	1		1	
Female	2.06 (1.68, 2.52)	**0.000**	2.10 (1.72, 2.58)	**0.000**
**Wealth quintile**				
Poor	1		1	
Poorer	0.71 (0.49, 1.01)	0.055	0.73 (0.50, 1.05)	0.090
Middle	1.01 (0.71, 1.45)	0.936	1.11 (0.76, 1.62)	0.586
Richer	0.98 (0.69, 1.39)	0.918	1.11 (0.75, 1.64)	0.589
Richest	2.32 (1.63, 3.31)	**0.000**	2.31 (1.58, 3.38)	**0.000**
**Daily Household food expenditure**				
< 4000 CFA	1		1	
> 4000 CFA	1.51 (1.17, 1.94)	**0.001**	1.32 (1.02, 1.72)	**0.033**
**Household food security level**				
Food Secure	1		1	
Food Insecure	0.91 (0.72, 1.15)	0.452	1.23 (0.94, 1.61)	0.135

*A three-level ordered logistic regression was estimated with individuals nested in households and households nested in study areas. Random intercepts were included at both the household and study area levels to account for clustering effects.*Bold values indicate statistical significance at p < 0.05

After adjusting for key household characteristics, age, sex, highest wealth status, and daily household food expenditure remained significant predictors of high BMI, as shown in Table 5. Age was a significant positive predictor of increased BMI in both crude and adjusted models (AOR = 1.13, 95% CI: 1.09, 1.17). Females were consistently more likely to have higher BMI compared to males (AOR = 2.10, 95% CI: 1.72, 2.58), highlighting substantial gender disparities. Notably, individuals belonging to the richest wealth quintile household were significantly more likely to have a high BMI (AOR = 2.31, 95% CI: 1.58, 3.38). Additionally, households spending more than 4000 CFA daily on food were significantly associated with high BMI status (AOR = 1.32, 95% CI: 1.02, 1.72). However, individuals from food-insecure households were still 1.20 times more likely to have high BMI, but this was not statistically significant.

**Table 6:** Adjusted multilevel regression model for the association between household characteristics and BMI by study area

	Boundou area		Bedik area		Senegal River area		Lower Casamance area		Dakar area	
	BMI AOR (95% CI)	*P* value	BMI AOR (95% CI)	*P* value	BMI AOR (95% CI)	*P* value	BMI AOR (95% CI)	*P* value	BMI	*P* value
**Age**	1.12 (1.04, 1.19)	**0.002**	0.98 (0.90, 1.08)	0.813	1.16 (1.05, 1.28)	**0.004**	1.18 (1.11, 1.27)	**0.000**	1.24 (1.11, 1.39)	**0.000**
**Sex**										
Male	1		1		1		1		1	
Female	2.03 (1.35, 3.04)	**0.001**	2.62 (1.54, 4.46)	**0.000**	1.68 (1.02, 2.75)	**0.040**	2.73 (1.79, 4.18)	**0.000**	2.07 (1.23, 3.45)	0.006
**Daily Household food expenditure**										
< 4000 CFA	1		1		1		1		1	
> 4000 CFA	2.13 (1.29, 3.53)	**0.003**	1.06 (0.56, 2.02)	0.856	0.96 (0.52, 1.79)	0.905	1.83 (0.86, 3.86)	0.569	1.09 (0.57, 2.08)	0.786
**Household food security level**										
Food secure	1		1		1		1		1	
Food Insecure	2.16 (1.32, 3.52)	**0.002**	0.92 (0.47, 1.81)	0.814	0.95 (0.56, 1.77)	0.987	0.72 (0.27, 1.88)	0.355	0.96 (0.52, 1.80)	0.911

*Bold values indicate statistical significance at *p* < 0.05

Table 6 presents the adjusted multilevel regression models assessing the association between household characteristics and BMI, stratified by study area. Across the study areas, age was significantly associated with higher BMI in Boundou, Senegal River, Lower Casamance, and Dakar, but not in Bedik. Females were consistently associated with significantly higher BMI compared to males in all regions. Higher daily household food expenditure was significantly associated with higher BMI only in Boundou (AOR = 2.13, 95% CI: 1.29, 3.53), while the effect was non-significant in other regions. Food insecurity was significantly associated with higher BMI only in Boundou (AOR = 2.16, 95% CI: 1.32, 3.52), while in all other regions, the association between food insecurity and BMI was weak and not statistically significant. This pattern highlights that while some determinants like sex and age show consistent effects across regions, the impact of food insecurity and household spending on BMI appears to be highly context-specific, with Boundou standing out as a rural area where food insecurity is paradoxically linked to higher BMI.

**Table 7:** Adjusted multilevel regression model for the association between household characteristics and categories of BMI

	Obese AOR (95% CI)	*P* value	Overweight AOR (95% CI)	*P* value	Underweight AOR (95% CI)	*P* value
Age	1.16 (1.08, 1.24)	**0.000**	1.11 (1.05, 1.73)	**0.000**	0.93 (0.87, 0.98)	**0.018**
**Sex**						
Male	1		1		1	
Female	3.83 (2.46, 5.97)	**0.000**	2.48 (1.82, 3.38)	**0.000**	1.17 (0.83, 1.66)	0.361
**Daily household food expenditure**						
< 4000 CFA	1		1		1	
> 4000 CFA	1.50 (1.02, 2.23)	**0.040**	1.41 (1.01, 1.96)	**0.044**	0.90 (0.55, 1.46)	0.675
**Household food security level**						
Food secure	1		1		1	
Food Insecure	1.21 (0.78, 1.86)	0.382	1.15 (0.82, 1.61)	0.406	0.72 (0.46, 1.16)	0.182
**Study area**						
Dakar	1		1		1	
Boundou	0.49 (0.27, 0.89)	**0.018**	0.53 (0.32, 0.85)	**0.009**	3.35 (1.54, 7.27)	**0.002**
Bedik	0.38 (0.21, 0.69)	**0.002**	0.38 (0.23, 0.62)	**0.000**	1.76 (0.82, 3.79)	**0.145**
Senegal River	0.27 (0.15, 0.53)	**0.000**	0.47 (0.29, 0.76)	**0.002**	1.01 (0.46, 2.22)	0.975
Lower Casamance	1.31 (0.76, 2.27)	0.337	0.98 (0.59, 1.63)	0.956	1.43 (0.63, 3.23)	0.387

*Bold values indicate statistical significance at *p* < 0.05

Table 7 presents the adjusted multilevel regression results examining the association between household characteristics and BMI categories (obesity, overweight, underweight) across different study areas. Age was positively associated with obesity and overweight and negatively associated with underweight. Females had significantly higher odds of being obese (AOR = 3.83, 95% CI: 2.46, 5.97) and overweight (AOR = 2.48, 95% CI: 1.82, 3.38) compared to males, though no significant association was observed for underweight. Households spending more than 4000 CFA daily on food were significantly associated with increased odds of obesity (AOR = 1.50, 95% CI: 1.02, 2.23), and overweight (AOR = 1.41, 95% CI: 1.01, 1.96) but not underweight. While the model suggested a slightly increased likelihood of obesity and overweight among food-insecure households, this association was not statistically significant. However, regional differences were notable. Compared to Dakar, individuals residing in Boundou, Bedik, and the Senegal River areas had significantly lower odds of being obese and overweight. However, individuals in Boundou had more than three times the odds of being underweight (OR = 3.35, 95% CI: 1.54, 7.27), suggesting a double burden of malnutrition across regions.

**Table 8:** Adjusted multilevel regression model for the association between household characteristics and overweight by study area

	Boundou area		Bedik area		Senegal River area		Lower Casamance area		Dakar area	
	Overweight AOR (95% CI)	*P* value	Overweight AOR (95% CI)	*P* value	Overweight AOR (95% CI)	*P* value	Overweight AOR (95% CI)	*P* value	Overweight	*P* value
**Age**	1.08 (0.97, 1.20)	0.146	1.03 (0.89, 1.19)	0.651	1.12 (0.99, 1.27)	0.062	1.18 (1.06, 1.32)	**0.002**	1.15 (0.99, 1.25)	0.053
**Sex**										
Male	1		1		1		1		1	
Female	2.55 (1.29, 5.03)	**0.007**	3.94 (1.39, 11.15)	**0.010**	2.73 (1.38, 5.39)	**0.004**	2.45 (1.31, 4.57)	**0.005**	2.03 (1.02, 4.05)	**0.044**
**Daily Household food expenditure**										
< 4000 CFA	1		1		1		1		1	
> 4000 CFA	1.67 (0.92, 3.06)	0.092	1.64 (0.71, 3.78)	0.250	0.74 (0.37, 1.48)	0.406	1.03 (0.37, 2.85)	0.956	2.64 (1.18, 5.89)	**0.018**
**Household food security level**										
Food secure	1		1		1		1		1	
Food Insecure	0.61 (0.33, 1.14)	0.125	1.17 (0.49, 2.75)	0.716	1.10 (0.58, 1.98)	0.803	0.70 (0.17, 2.82)	0.624	2.16 (1.04, 4.45)	**0.038**

*Bold values indicate statistical significance at *p* < 0.05

Table 8 presents adjusted multilevel regression results for the association between household characteristics and overweight status across five study areas. Sex was a strong and consistent predictor of being overweight in the region, with women having significantly higher odds than men. Daily household food expenditure showed a significant association with overweight only in Dakar, where households spending more than 4000 CFA were 2.64 times more likely to be overweight. Food insecurity was not significantly associated with being overweight in most regions, except in Dakar, where food-insecure individuals were more than twice as likely to be overweight (AOR = 2.16, 95% CI: 1.04, 4.45).

**Table 9:** Adjusted multilevel regression model for the association between household characteristics and underweight by study area

	Boundou area		Bedik area		Senegal River area		Lower Casamance area		Dakar area	
	Underweight AOR (95% CI)	*P* value	Underweight AOR (95% CI)	*P* value	Underweight AOR (95% CI)	*P* value	Underweight AOR (95% CI)	*P* value	Underweight AOR (95% CI)	*P* value
**Age**	0.95 (0.87, 1.04)	0.261	1.03 (0.87, 1.21)	0.750	0.81 (0.59, 1.11)	0.184	0.93 (0.84, 1.04)	0.215	0.89 (0.72, 1.11)	0.301
**Sex**										
Male	1		1		1		1		1	
Female	0.78 (0.46, 1.32)	0.199	1.58 (0.65, 3.88)	0.947	1.99 (0.43, 9.33)	0.380	1.05 (0.52, 2.12)	0.893	2.43 (0.77, 7.71)	0.129
**Daily Household food expenditure**										
< 4000 CFA	1		1		1		1		1	
> 4000 CFA	0.45 (0.21, 0.96)	**0.038**	1.66 (0.49, 5.52)	0.314	2.35 (0.27, 20.9)	0.406	0.61 (0.16, 2.27)	0.459	1.98 (0.56, 6.92)	0.287
**Household food security level**										
Food secure	1		1		1		1		1	
Food Insecure	0.24 (0.13, 0.43)	**0.000**	2.67 (0.73, 9.88)	0.140	1.97 (0.22, 17.7)	0.546	1.95 (0.49, 7.71)	0.341	1.75 (0.57, 5.32)	0.325

*Bold values indicate statistical significance at *p* < 0.05

Table 9 presents the adjusted multilevel regression results examining the association between household characteristics and underweight status across different study areas. Overall, age and sex were not significantly associated with underweight in any region. However, in Boundou, two household-level factors emerged as significant, as individuals from households spending more than 4000 CFA daily on food had significantly lower odds of being underweight (OR = 0.45, 95% CI: 0.21, 0.96) and surprisingly food-insecure households were also less likely to have underweight adults (OR = 0.24, 95% CI: 0.13, 0.43). In contrast, in Bedik, Senegal River, Lower Casamance, and Dakar, none of the household-level variables, including food expenditure and food security, were found to be significantly associated with underweight.

## Discussion

The present study examined the association between household food insecurity and the nutritional status of adults across five regions in Senegal. The findings highlighted significant regional disparities in food security and nutritional outcomes, underscoring the complexity of food insecurity and its impact on body mass index (BMI). The results of this study revealed a high prevalence of food insecurity in the Boundou and Bedik/Bandafassi areas, where over 50% of households were severely food insecure, and in the Senegal River area, about 37% of households were also severely food insecure. In contrast, Lower Casamance had the highest proportion of food-secure households. This regional variation aligns with previous studies, which indicate that geographic and economic factors influence food availability and access [9]. Casamance’s high food security may be attributed to its fertile floodplain landscape, which supports rice agriculture and fisheries, ensuring relatively stable food availability. In contrast, the semi-arid environments of Boundou and Bedik/Bandafassi, characterized by pastoralism and rainfed agriculture, are more vulnerable to climate variability, such as droughts and heatwaves, which exacerbate food insecurity. Recent analyses of food systems in sub-Saharan Africa highlight that climate variability, including recurrent droughts and rainfall instability, continues to intensify food insecurity in semi-arid and rainfed agricultural zones, particularly in Sahelian regions [29, 30]. In Senegal, eastern dryland regions remain particularly vulnerable due to limited irrigation infrastructure and dependence on climate-sensitive livelihoods [31, 32]. Our findings also corroborate those of another study, which reported that vulnerability to food security in Senegal varies by geographic region, with food insecurity more prevalent in the eastern parts of Senegal, such as Boundou and Bedik/Bandafassi [33]. It is important to note that food insecurity in this study was measured using the Household Food Insecurity Access Scale (HFIAS), a tool previously validated in several low- and middle-income settings, including sub-Saharan Africa [25, 26]. While the HFIAS captures behavioural and experiential aspects of food insecurity, we acknowledge that regional cultural nuances may influence responses. Nevertheless, the tool remains a widely accepted method for cross-contextual comparisons and provides valuable insight into household access to food in the Senegalese context.

The regional variations in nutritional outcomes observed in this study highlighted the complexity of the food insecurity-nutrition relationship across Senegal. Boundou and Bedik/Bandafassi regions had the highest prevalence of underweight individuals, while Dakar and Lower Casamance had higher rates of overweight and obese individuals. One might imagine a household in Boundou, scraping together daily meals of millet and sorghum, illustrating commonly documented rural subsistence diets [22, 34], while another in Dakar, though not necessarily affluent, purchases fast food and sugary drinks from nearby kiosks [35, 36]. We therefore argue that this pattern reflects the dual burden of malnutrition, whereby undernutrition and overnutrition coexist within the same country or even the same households, driven by regionally distinct food environments and stages of nutrition transition. The high prevalence of underweight individuals in Boundou and Bedik/Bandafassi could suggest that chronic food shortages and poor dietary diversity may be key contributors to malnutrition in these areas. Furthermore, the high prevalence of overweight and obesity in Dakar and Lower Casamance, despite relatively higher levels of food security in the households, may also reflect the influence of urbanization and changing dietary patterns. Urban food systems research in sub-Saharan Africa shows that rapid urbanization alters food environments and dietary behaviours, increasing access to processed and energy-dense products, thereby contributing to evolving nutrition outcomes amid ongoing food security challenges [22, 37–39]. Notably, there was also underweight present in the Lower Casamance region. This double burden, as seen in Senegal, reflects the country’s ongoing nutrition transition. The Senegalese region is experiencing a nutrition transition [22], and urban areas now have greater access to processed and fast foods, which are typically high in calories, sugar, and fat but low in essential nutrients [11]. This shift towards unhealthy diets, combined with reduced physical activity, may explain the higher rates of overweight and obesity in these regions. Interestingly, even in Lower Casamance, where food security was high, a notable proportion of adults were underweight, underscoring the importance of considering factors beyond food availability, such as illness, micronutrient deficiencies, or unequal intra-household food distribution in understanding nutritional outcomes.

The study revealed significant associations between household characteristics and having a higher BMI. Age was a significant predictor of BMI, with older individuals more likely to be overweight or obese. In addition, our study found that female participants had significantly higher odds of being overweight or obese compared to males, with women being two times more likely to have a higher BMI. This finding is widely supported in the literature, as studies across Africa and other regions have shown that women have the highest risk of being overweight or obese [40–42]. Social norms and roles may influence women’s dietary behaviors and physical activity patterns differently from those of men, often leading to higher BMI outcomes among women [43]. In Senegalese society, for instance, body fat is culturally associated with beauty, status, or wealth, particularly in women, further complicating public health messaging around healthy weight. Beyond sociocultural norms, structural gender disparities also shape nutritional outcomes. Women across West Africa continue to face limited access to land ownership, irrigation infrastructure, and agricultural inputs, constraining their economic autonomy and dietary decision-making power [44, 45]. After adjusting for confounders, individuals from households in the richest wealth quintile were two times more likely to have a higher BMI than those in the poorest quintile. These findings are consistent with global evidence suggesting that economic affluence can be linked to an increased BMI, likely due to greater access to processed foods and sedentary lifestyles [46, 47]. One could argue that wealth is likely to underlie some of the regional contrasts, as the relatively affluent populations of Lower Casamance and Dakar have a higher prevalence of obesity and overweight individuals, suggesting that poverty remains a significant barrier to adequate nutrition in some regions of Senegal.

Daily household food expenditure was also associated with a higher BMI, with households spending more than 4000 CFA on food having significantly higher odds of individuals with an increased BMI. This finding underscored the importance of economic access to food in shaping nutritional outcomes. However, it also highlighted the need to consider the quality of food consumed, as higher expenditures do not necessarily translate into healthier diets or increased diversity of food [3, 22].

In this study, the relationship between food insecurity and adult BMI was not straightforward. Although the association was not statistically significant in the overall model (Table 5), the trends suggest that adults from food-insecure households may be at increased risk of overweight and obesity. This supports previous evidence suggesting that food insecurity and obesity coexist in disadvantaged households in developing countries [48, 49]. Crucially, we found that the relationship between household food insecurity and adult BMI was highly context-specific, as the region-stratified analyses (Table 6) revealed an intriguing divergence. In Boundou, household food insecurity was positively associated with a higher BMI, whereas in other regions, no such relationship was observed. Likewise, in the specific region-stratified model for overweight status (Table 8), food insecurity was a significant risk factor only in urban Dakar, where food-insecure individuals were more than twice as likely to be overweight. The Boundou finding was particularly notable. Despite being the region with the most severely food-insecure households, food-insecure individuals tended toward higher BMI and had lower odds of being underweight. This finding, to an extent, supports the “food insecurity-obesity hypothesis,” which has been documented in both high-income and low-income countries [5, 6, 50, 51]. One of the mechanisms proposed in the “food insecurity-obesity paradox” is that food insecurity promotes the purchase of energy-dense foods for household consumption, meaning that food-insecure individuals may rely on cheaper, calorie-dense foods that provide sufficient energy but lack essential nutrients, leading to weight gain and poor nutritional quality even amidst poverty [39, 52, 53].

In urban Dakar, there was also an observed positive link between food insecurity and overweight. We argue that, in Lower Casamance, which is still traditional, obesity may still be predominantly associated with wealth, but in urban areas, there might be a transition to cheap food, making poor people fat, whereas in Senegal River is the other way round where food insecure people are underweight and these diverse associations underscore that the nutrition transition in Senegal is occurring in distinct phases, reflecting unique regional economic and sociocultural dynamics. Urban poor often have access to inexpensive, nutrient-poor processed foods, along with sedentary lifestyles [13, 54, 55]. Our finding that food-insecure adults in Dakar were about two times more likely to be overweight than food-secure ones aligns with studies in other low-income urban African settings, which have reported that individuals experiencing food insecurity tend to have higher weight gain, leading to overweight or obesity [56–58]. Stress-related eating, consumption of sugar-sweetened beverages, and reduced physical activity in city slums can contribute to this paradox [59, 60]. As reported in another study conducted in Senegal, food-insufficient households were purchasing and consuming high-calorie foods such as sugar, cooking oil, fats, and fried foods [22]. Moreover, in our study areas, food consumption has changed dramatically due to the ongoing nutrition transition that has occurred in Senegal, and these changes have been influenced by different factors, including institutional changes, environmental and economic evolutions, as well as demographic, social, and cultural trends [22, 61]. Furthermore, in this present time in Senegal, globalized food chains have contributed to the increased supply of processed foods high in sugar, salt, and oil that are cheaper to produce and transport.

The understanding of the coexistence of both food insecurity and obesity/overweight, although it might seem contradictory, is relevant for generating further public health actions. It is important to note that research on the relationship between household food insecurity and BMI has produced mixed results. Some studies have reported a positive association between household food insecurity and obesity/overweight [5, 62], while others have found a negative or no association [63–65] with obesity/overweight among adults. In addition, that food insecurity and obesity can co-exist and are significantly associated in some studies does not necessarily mean they are causally linked to each other. Both food insecurity and obesity can be independent consequences of low income and the resulting lack of access to enough nutritious food or poverty. In fact, obesity is a chronic condition that develops over months to years, and food insecurity can be either periodic or chronic. Therefore, because individuals experience these conditions over time, their association cannot necessarily be explained by a study in which each is measured at only one point in time. Thus, we suggest further research to explore this complex issue.

The underweight-related findings from this study reveal a complex and regionally variable landscape of adult undernutrition in Senegal, shaped by both individual-level characteristics and broader ecological contexts. From the national multilevel regression model (Table 7), age emerged as a significant predictor, with younger adults more likely to be underweight. This suggests that across the national sample, younger adults are more vulnerable to chronic energy deficiency. While we expected a strong association between food insecurity and underweight status in the national multilevel regression model, our findings did not support this hypothesis. One possible explanation is that even food-insecure households prioritize caloric sufficiency over dietary diversity, consuming staple foods that provide enough energy but lack essential micronutrients [66, 67]. In other words, in contexts of food insecurity, people facing food insecurity may have to compromise on food quantity and quality, often consuming less diverse diets and turning to cheaper, calorie-dense foods to alleviate hunger. While this approach may prevent underweight, it still leads to inadequate intake of essential nutrients, resulting in poor nutritional status. This aligns with previous research indicating that underweight may be driven more by severe poverty and health conditions rather than food insecurity alone [68]. Adults living in Boundou were found to have significantly higher odds of being underweight compared to adults living in Dakar. This result for Boundou is consistent with the descriptive data in Table 4, which shows that the area had the highest prevalence of underweight adults in the sample. These findings reinforce the characterization of the Boundou area, located in the eastern region of Senegal, as one of the most food-insecure and ecologically fragile zones in the country, a dryland region reliant on pastoralism and subsistence agriculture, both of which are highly vulnerable to climate variability, seasonal food gaps, and infrastructural limitations. Recent studies have highlighted that land use constraints, limited water access, and insecure land tenure in dryland regions can exacerbate vulnerability to food insecurity and undernutrition, particularly among pastoralist and subsistence farming communities [69–73]. Furthermore, the significantly elevated risk of underweight in Boundou reflects the cumulative impact of chronic food insecurity, poverty, and limited dietary diversity, which contribute to persistent energy deficiency among adults. It is important to note that while Lower Casamance also had relatively high underweight prevalence of 14% despite the high level of food security in the region, their adjusted odds national multilevel regression model was not statistically different, suggesting that factors beyond geographic location could be playing a role in the nutritional outcomes in this area.

## Strengths and limitations of the study

This study has several notable strengths. First, the large sample across five ecologically distinct regions of Senegal enabled both pooled and region-stratified analyses, providing a rare opportunity to examine how the relationship between household food insecurity and adult nutritional status varies across socioecological contexts within a single country. The results of the current study should nonetheless be interpreted in light of several limitations. Firstly, the cross-sectional nature of the data limits our ability to draw any causal conclusions about the relationship between household food insecurity and adult nutritional status, particularly given that obesity develops over months to years while food insecurity may be either periodic or chronic. Secondly, this study was conducted during April and June, therefore, it does not capture the influence of seasonality on food insecurity and malnutrition. The prevalence of food insecurity and malnutrition is expected to be higher during the lean season, which lasts from the end of June to September in Senegal, corresponding to the period between harvests. Finally, the HFIAS, although a validated instrument, relies on self-report and may therefore be subject to recall or social desirability bias. Despite these limitations, our results have shed more light on the association between household food insecurity and the nutritional status of adults in LMICs such as Senegal, which should also be of use in designing future research.

## Conclusion

This study provided empirical evidence demonstrating that the relationship between household food insecurity and adult nutritional status in Senegal was highly context-specific and regionally differentiated, with the most notable association observed in Boundou region, where food insecurity was linked to higher BMI, despite it being the most food-insecure region, a finding that supports the food insecurity-obesity hypothesis. The coexistence of high underweight prevalence in Boundou and Bedik/Bandafassi alongside elevated overweight and obesity rates in Dakar and Casamance illustrates Senegal’s evolving double burden of malnutrition within distinct socioecological environments. The study’s findings indicate that national nutrition strategies must be tailored to local realities. In dryland rural regions such as Boundou, policy attention should focus on improving food access, income generation, and health system strengthening to address both dietary quantity and quality. Investment in climate-resilient agricultural systems and stronger land tenure security, particularly for smallholders and women, may further enhance agricultural productivity, diversification, and dietary diversity. In contrast, in regions like Dakar and Casamance, a more multi-sectoral approach may be required, one that includes health screening, dietary education, and poverty reduction strategies to address both overweight and hidden forms of undernutrition not captured by food access alone. Given that food insecurity may act as a predictor of overweight and obesity, policies and interventions aimed at reducing food insecurity should simultaneously address the multiple burdens of malnutrition. However, it is important to recognize that malnutrition is not solely a matter of food availability or intake, as health is shaped by the broader societal and economic context in which an individual lives. Addressing Senegal’s malnutrition burden therefore requires coordinated action across agricultural, health, and economic sectors that recognizes the broader structural determinants shaping nutritional outcomes.

## Supplementary Information

Below is the link to the electronic supplementary material.

Supplementary Material 1

## Data Availability

The datasets used and/or analyzed during the current study are available from the corresponding author on reasonable request.

## Abbreviations

AIC: Akaike information criterion
ANSD: Agence nationale de la statistique et de la Démographie
AOR: Adjusted odds ratio
BMI: Body mass index
CI: Confidence interval
CNERS: National ethics committee for health research in Senegal
CFA: Communauté Financière Africaine (West African CFA franc)
DHS: Demographic and health survey
EKNZ: Ethikkommission Nordwest–und Zentralschweiz
FAO: Food and agriculture organization of the United Nations
HFIAS: Household food insecurity access scale
IFAD: International fund for agricultural development
IFAN: Institut Fondamental d’Afrique Noire
LMICs: Low–and niddle–income countries
OR: Odds ratio
SES: Socioeconomic status
SNF: Swiss national science foundation
SSA: Sub–Saharan Africa
UCAD: Université cheikh anta diop
UNICEF: United nations children’s fund
USAID: United States agency for international development
WHO: World health organization
WFP: World food programme
