# Supplementary material for: Nutrition status among adults in the face of household food insecurity: a cross-sectional study in five different regions in senegal
Source: Agric Food Secur. 2026 Jun 14;15(1):62. doi: 10.1186/s40066-026-00643-7 (PMC13493398; doi:10.1186/s40066-026-00643-7)
Supplement: Supplementary file 1 [file 40066_2026_643_MOESM1_ESM.docx]

**Supplementary Table S1**

| **Section 5: Household Food Insecurity Access Scale** | | | |
| --- | --- | --- | --- |
| 5.01 | In the past four weeks, did you worry that your household would not have enough food? | 0 = No (skip to Q2) 1=Yes |  |
| 5.01a | How often did this happen? | 1 = Rarely (once or twice in the past four weeks)  2 = Sometimes (three to ten times in the past four weeks)  3 = Often (more than ten times in the past four weeks) |  |
| 5.02 | In the past four weeks, were you or any household member not able to eat the kinds of foods you preferred because of a lack of resources? | 0 = No (skip to Q3) 1=Yes |  |
| 5.02a | How often did this happen? | 1 = Rarely (once or twice in the past four weeks)  2 = Sometimes (three to ten times in the past four weeks)  3 = Often (more than ten times in the past four weeks) |  |
| 5.03 | In the past four weeks, did you or any household member have to eat a limited variety of foods due to a lack of resources? | 0 = No (skip to Q4) 1=Yes |  |
| 5.03a | How often did this happen? | 1 = Rarely (once or twice in the past four weeks)  2 = Sometimes (three to ten times in the past four weeks)  3 = Often (more than ten times in the past four weeks) |  |
| 5.04 | In the past four weeks, did you or any household member have to eat some foods that you really did not want to eat because of a lack of resources to obtain other types of food? | 0 = No (skip to Q5) 1=Yes |  |
| 5.04a | How often did this happen? | 1 = Rarely (once or twice in the past four weeks)  2 = Sometimes (three to ten times in the past four weeks)  3 = Often (more than ten times in the past four weeks) |  |
| 5.05 | In the past four weeks, did you or any household member have to eat a smaller meal than you felt you needed because there was not enough food? | 0 = No (skip to Q6) 1=Yes |  |
| 5.05a | How often did this happen? | 1 = Rarely (once or twice in the past four weeks)  2 = Sometimes (three to ten times in the past four weeks)  3 = Often (more than ten times in the past four weeks) |  |
| 5.06 | In the past four weeks, did you or any other household member have to eat fewer meals in a day because there was not enough food? | 0 = No (skip to Q7) 1=Yes |  |
| 5.06a | How often did this happen? | 1 = Rarely (once or twice in the past four weeks)  2 = Sometimes (three to ten times in the past four weeks)  3 = Often (more than ten times in the past four weeks) |  |
| 5.07 | In the past four weeks, was there ever no food to eat of any kind in your household because of lack of resources to get food? | 0 = No (skip to Q8) 1=Yes |  |
| 5.07a | How often did this happen? | 1 = Rarely (once or twice in the past four weeks)  2 = Sometimes (three to ten times in the past four weeks)  3 = Often (more than ten times in the past four weeks) |  |
| 5.08 | In the past four weeks, did you or any household member go to sleep at night hungry because there was not enough food? | 0 = No (skip to Q9) 1=Yes |  |
| 5.08a | How often did this happen? | 1 = Rarely (once or twice in the past four weeks)  2 = Sometimes (three to ten times in the past four weeks)  3 = Often (more than ten times in the past four weeks) |  |
| 5.09 | In the past four weeks, did you or any household member go a whole day and night without eating anything because there was not enough food? | 0 = No  1=Yes |  |
| 5.09a | How often did this happen? | 1 = Rarely (once or twice in the past four weeks)  2 = Sometimes (three to ten times in the past four weeks)  3 = Often (more than ten times in the past four weeks) |  |
